# Experimental Study on Compressive Behavior of Concrete Cylinders Confined by a Novel Hybrid Fiber-Reinforced Polymer Spiral

**DOI:** 10.3390/polym14214750

**Published:** 2022-11-05

**Authors:** Yu Tang, Xiaowan Lu, Yang Wei, Shitong Hou

**Affiliations:** 1 College of Civil Engineering, Nanjing Forestry University, Nanjing 210037, China; 2 College of Civil Engineering, Southeast University, Nanjing 210096, China

**Keywords:** compressive behavior, concrete cylinder, FRP spiral, stress–strain relationship

## Abstract

Modern fiber-reinforced polymer (FRP)-reinforced concrete structures are excepted to achieve superior mechanical performances and long service lives, even in harsh service environments. Hybrid FRP material could potentially meet this goal with its relatively high strength-to-cost ratio. This paper presents an experimental study on the compressive behavior of concrete cylinders confined by a novel hybrid fiber-reinforced polymer (HFRP) spiral. Nine types, forming a total of 27 confined or non-confined concrete cylinders, were subjected to an axial compressive-loading test. Concrete cylinders confined either with different spiral types or different spiral spacings were comparatively studied in the experiment. The results showed that the compressive failure modes and the stress–strain relationships of the HFRP-spiral-confined cylinders were similar to those of basalt-fiber-reinforced polymer (BFRP)-spiral-confined cylinders. The actual fracture strain of the HFRP spiral (tested as a single rod) was larger than that of the corresponding carbon-fiber-reinforced polymer (CFRP) bar, indicating the advantageous composite effect of the HFRP spiral. The maximum strain of the HFRP spiral reached over 70% of its ultimate strain in the cylinders compared to the BFRP spiral, which only reached 50%. Most of the existing models overestimated the ultimate stress and strain of the HFRP-spiral-confined cylinders. Wu’s model was proved to be the most accurate model, yet proper modification was required for predicting the peak strain of the HFRP-confined cylinders.

## 1. Introduction

Fiber-reinforced polymer (FRP) composites have been widely used in concrete structures due to their superior anticorrosion properties and high strength-to-weight ratios [1]. Applying FRP composites to the hoop constraints of concrete columns is a generally accepted application scenario to reach full strength potential, which is considered to be beneficial to enhance the compressive strength and ultimate strain of concrete columns significantly [2]. As a result, a durable and cost-effective design could be achieved by FRP-confined concrete structures.

**Figure 1:**
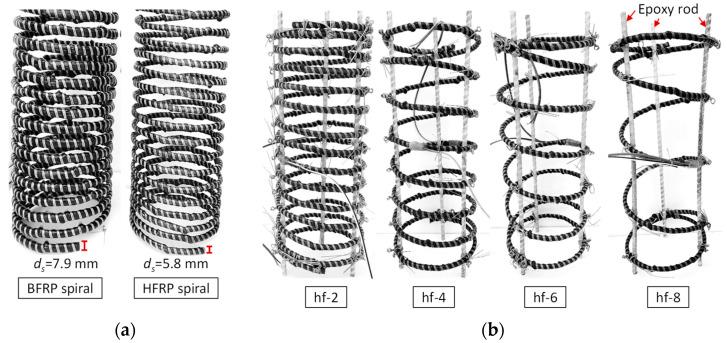
Different spirals and reinforcement cages in test cylinders: (**a**) different spirals; (**b**) reinforcement cages with different spacings.

In early applications, due to their relatively high cost FRP composites were usually introduced in retrofitting technologies for repairing or strengthening concrete structures. FRP sheets and strips were commonly used to wrap concrete columns to achieve a strong constraint effect for the columns and to enhance their compressive performances [3,4]. With costs reducing and demand growing, more types of novel FRP products have been developed recently, such as the FRP tube [5], FRP grid [6], FRP steel composite tube [7], etc. These products have expanded the application scenarios of FRP composites in civil structures, for example, to high-rise and large-span structures [8], tunnel structures [9], marine structures [10,11], etc. During the retrofitting process for certain underwater structures (such as bridge piers or dams), traditional externally bonded FRP sheets and strips are inapplicable under these scenario due to the underwater environment restricting the application of most bonding materials. In addition, to achieve a long service life for retrofitted underwater structures potentially subject to harsh service environments, an embedded FRP product, such as an FRP spiral (in Figure 1a) or FRP grid, is preferred.

In terms of fiber type (for composing FRP spirals or grids), there are roughly two types of fibers, which either pursue maximum strength or stiffness (as carbon fiber [12]) but carry a relative deformation ability or pursue the maximum deformation ability (as basalt fiber [13], glass fiber [14], PET fiber [15,16], etc.) with relatively low strength. The strength or stiffness ability of a fiber can be beneficial to its constraint efficiency for confined concrete [17], while its deformation ability can also be in favor of enhancing the seismic performances of concrete structures [18]. Incorporating the advantages of different types of fibers while easing their disadvantages as much as possible during application is an important focused question.

From an operational level, an active confinement method [19] is a potentially effective way for certain fibers with a relatively low stiffnesses (but with relatively high deformation abilities). From a material innovation level and considering the extreme performance differences between these two types of fibers, developing novel FRP composites that are composed of multiple layers or multiple types of fibers is a feasible way to integrate the advantages of different fibers. Multilayer FRP composites, such as steel FRP composite bars (SFCBs) [20,21] and FRP-steel composite tubes (CFCTs) [22], have been developed and studied for over a decade. Superior composite effects have been discovered in early studies [23,24], such as the post-yield stiffness of SFCBs, which was considered to be able to provide controllable post-yield deformation ability and reduce residual displacement for concrete structures [25]. Based on a similar mechanism, hybrid FRP materials composed of multiple types of fibers are supposed to develop the same advantageous composite effects as multilayer FRP composites. They may also be favorable for enhancing the mechanical performances of concrete structures with relatively low cost and even better anticorrosion performances (compared to certain multilayer FRP composites with exposed steel sections). However, this type of hybrid FRP material has rarely been developed and studied before.

Other than operational innovation and material innovation for FRP composites, many studies have focused on their design models in concrete structures. For instance, numerous compressive stress–strain models have been proposed for concrete columns confined by common FRP products, such as FRP sheets or FRP tubes composed of carbon fiber or glass fiber [2]. However, the applicability of these models is still unclear for novel FRP composites.

This paper presents an experimental study on the compressive behavior of concrete cylinders confined with a novel hybrid FRP (HFRP) spiral. The manufacturing method and mechanical properties of the HFRP spiral are first provided. Nine types, forming a total of 27 confined concrete cylinders, are subjected to an axial compressive-loading test. The effects of the FRP spiral type and spacing on the compressive behavior of the confined concrete cylinders are thoroughly analyzed and discussed. Several representative existing compressive stress–strain models of FRP-confined concrete are collected and evaluated with the test results. Modifications are suggested for a certain existing model to improve its accuracy in predicting the compressive performances of confined cylinders.

## 2. Experimental Program

### 2.1. Specimen Design

There were three series of cylinders with different reinforcements designed in the test: (1) plain concrete cylinders (pc); (2) basalt-fiber-reinforced polymer (BFRP) spiral (Figure 1a) reinforced cylinders (bf); and (3) hybrid fiber-reinforced polymer (HFRP) spiral (Figure 1a) reinforced cylinders (hf). Details of all the types of cylinders are listed in Table 1, where *s* refers to the spiral spacing; *ρ_f_* refers to the transverse reinforcement ratio; *f_cp_* and *ε_cp_* refer to the compressive peak stress and strain of a cylinder with strain-softening characteristics, respectively; and *f_cc_* and *ε_cc_* refer to the compressive ultimate stress and strain of a cylinder with strain-hardening characteristics, respectively.

For cylinder type in Table 1, hf-2 refers to the HFRP-spiral-reinforced cylinder with a spiral spacing of 23 mm; hf-4, hf-6, and hf-8 refer to the HFRP-spiral-reinforced cylinders with 2, 3, and 4 times the spiral spacing of the hf-2 cylinder, respectively. For comparison, corresponding BFRP-spiral-reinforced cylinders (bf-2, bf-4, bf-6, and bf-8) were also designed in the test. Each type of cylinder contained three identical specimens. For example, hf-2-1 refers to the first tested specimen of the three identical specimens.

**Figure 2:**
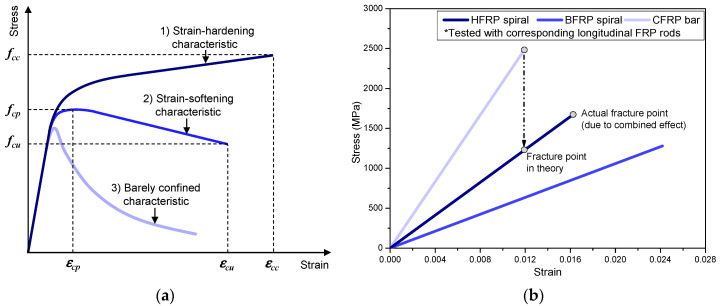
Confinement design principle and tensile properties of spirals: (**a**) confinement design principle; (**b**) tensile properties of different spirals.

The reinforcement details of the hf and bf cylinders are shown in Figure 1b. Different spacings of spirals were designed to achieve different constraint effects for the cylinders. There were three characteristic stress–strain relationships examined for the FRP-confined concrete, i.e., (1) stress–strain relationships with strain-hardening characteristics; (2) stress–strain relationships with strain-softening characteristics; and (3) barely confined characteristics (with almost no constraint effect). These are shown in Figure 2a, where *f_cu_* and *ε_cu_* refer to the compressive ultimate stress and strain of a cylinder with strain-softening characteristics, respectively. These characteristics were mainly controlled by the confinement strength and stiffness, which could vary with spiral spacing and elastic modulus, as designed in the current test. The thickness of the concrete cover of the reinforced cylinders (*d_c_*) was 15 mm. To fix the position of the HFRP (or BFRP) spirals in cylinders, three epoxy rods (diameter = 3 mm, fabricated with vinyl epoxy resin) were used as longitudinal reinforcement, as shown in Figure 1b. Their compressive contribution (to the cylinder) was neglected due to their minute cross-sectional area and brittle fracture feature.

The height and diameter (*D*) measurements of the cylinders were 300 mm and 150 mm, respectively [26,27]. To avoid local failure at the top and bottom ends of the cylinders, both ends were wrapped in two layers of CFRP sheets (width = 25 mm).

### 2.2. Material Properties

The HFRP spirals were composited with 20 bundles of 12 k carbon fiber, 22 bundles of 1200 tex basalt fiber, and vinyl epoxy resin. The BFRP spirals were composited with 82 bundles of basalt fiber and vinyl epoxy resin. The mass ratio of the fibers was approximately 85% of the FRP spirals. The mechanical and physical properties of spiral components are listed in Table 2, in which all values are the minimum values provided by the manufacturer.

The mechanical and physical properties of spirals are provided in Table 3 and Figure 2b, where *d_f_* refers to the diameter of the spiral; *A_f_* refers to the cross-sectional area of the spiral; *f_f_* and *E_f_* refer to the tensile strength and elastic modulus of the spiral, respectively; and *ε_fu_* refers to the ultimate strain of the spiral. The characteristic strength and modulus values of the spirals are the minimum values provided by the manufacturer. Since the spirals could not be directly tested (under axial tension), their strength and modulus values (in Table 3) were indirectly obtained through a tensile test (according to ASTM D7205M-06 [28]) with corresponding longitudinal HFRP (or BFRP) rods, which contained the same components as the spirals. However, due to possible fiber kinking in the spirals [29] (generated from manufacturing and application processes), their actual in-service mechanical properties could decrease.

Due to the possibility of confinement stiffness significantly affecting the compressive characteristics of FRP-reinforced concrete [30], the HFRP and BFRP spirals were designed with similar section stiffnesses (*E_f_* ·*A_f_* in Table 3) for a better comparison.

The concrete cylinders were cured in a natural environment for 28 days before the test. The elastic modulus and compressive strength values of the concrete (*f_co_′*) were 29 GPa (standard deviation = 1.7) and 44 MPa (standard deviation = 2.6), respectively. These were the average values tested according to ASTM C469-14 [31] and ASTM C39M-21 [32], respectively.

### 2.3. Test Instruments and Setup

**Figure 3:**
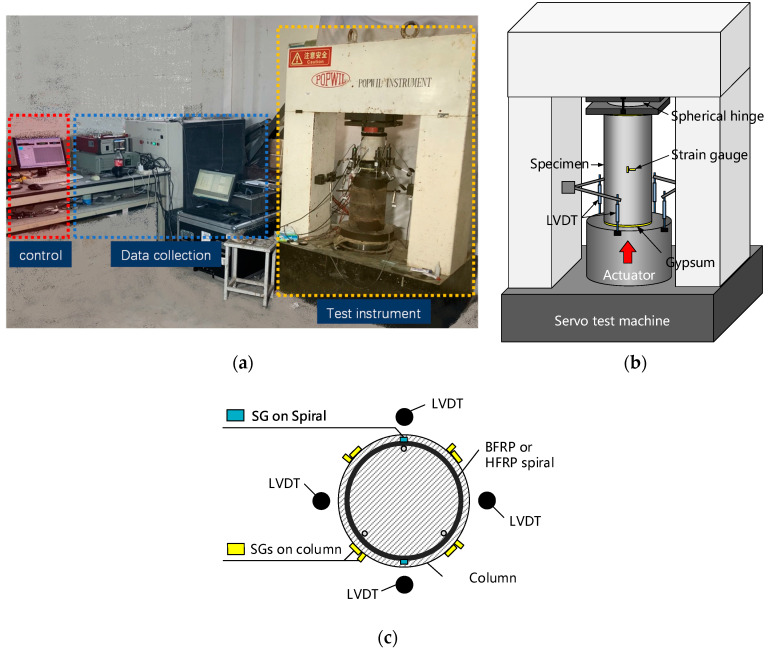
Test setup: (**a**) arrangement of test site; (**b**) test instrument; (**c**) sensor settings.

The arrangement of the test site is shown in Figure 3a. The compressive test was carried out on a servo compression machine, as shown in Figure 3b. A thin gypsum cushion was applied on the top and bottom surfaces of the cylinders to eliminate the gap between the cylinders and the test machine. The loading strain speed equaled 0.0012 per minute.

Four linear variable differential transformers (LVDTs) (stroke = 25 mm) evenly distributed at 90° around the cylinders were used to capture the loading displacements, as shown in Figure 3b. Their mean value was used to calculate the axial strain of the test cylinders. The axial stress was calculated with the loading force (directly obtained from the test machine) divided by the cylinder height.

The spiral strain was monitored with two strain gauges (SGs) (gauge length = 2 mm) pasted on the outward surfaces of the spirals and evenly distributed at 180° on the mid-height section of the cylinders, as shown in Figure 3c. Note that only one strain gauge (SG) was used to capture the spiral strain of the hf-8- and bf-8-type cylinders due to their large spiral spacing, which restricted the installation site of the SGs at the mid-height section of the cylinder. The axial and hoop strains of the cylinders were monitored with four axial and transverse SGs placed around the circumference of the cylinders (evenly spaced at 90° at mid-height), as shown in Figure 3c.

## 3. Test Results and Discussion

### 3.1. Stress–Strain Curves and Failure Modes of Cylinders

**Figure 4:**
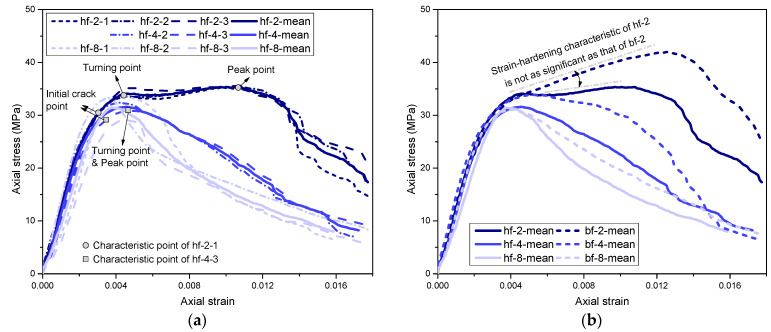
Axial stress–axial strain curves of different types of cylinders: (**a**) hf series; (**b**) comparison of hf series and bf series.

Table 1 lists the test results, which are the averaged mean values of three specimens for each type of cylinder. The typical axial stress–strain curves of the hf series cylinders are shown in Figure 4a. In addition, mean stress–strain curves averaged from three stress–strain curves for each type of cylinder are provided in Figure 4a (as “hf-2-mean”, “hf-4-mean”, and “hf-8-mean”). Particularly, the stress–strain curves of each type of column were first normalized with the same loading strain step (0.01). Then, the mean stress value (at each loading strain step) was averaged from the stress values of three specimens. Note that the stress–strain curve of the hf-4-1 curve was missed due to an unexpected test failure. The curve of the hf-4-mean in Figure 4, as well as the test results of the hf-4 cylinder in Table 1, were averaged only from hf-4-2 and hf-4-3.

(1) hf-2 cylinder type

**Figure 5:**
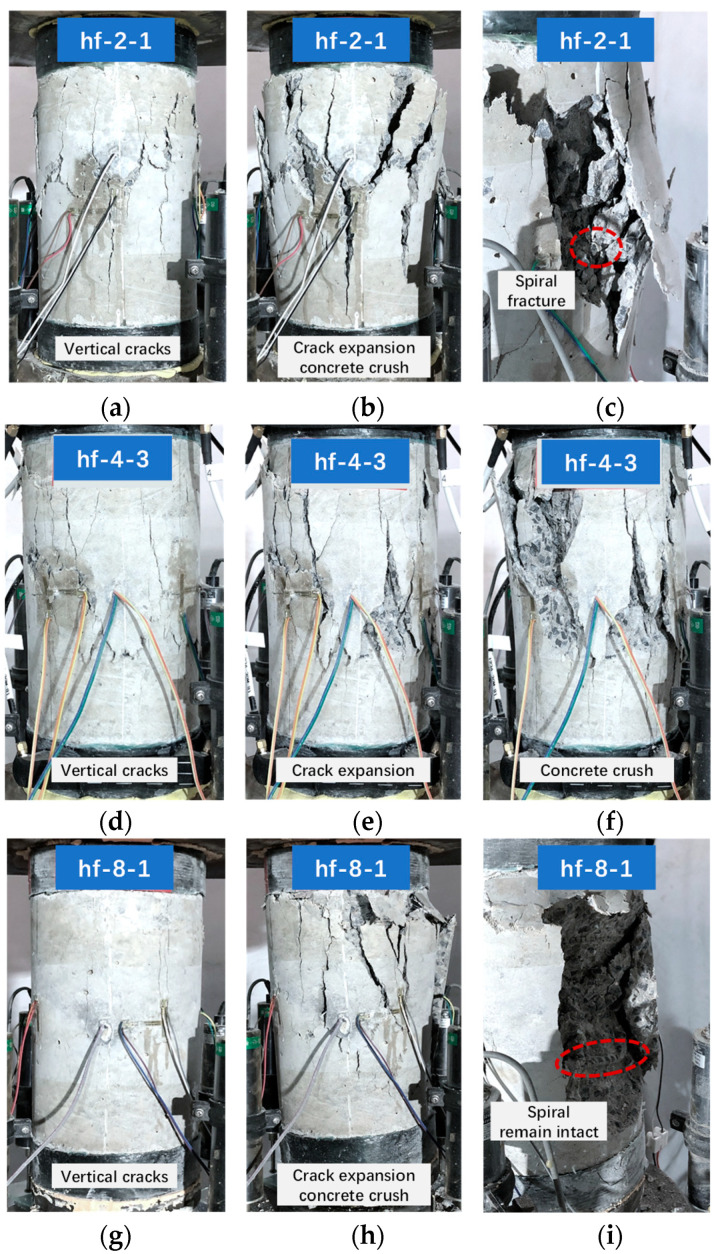
Typical failure process of hf series cylinders: (**a**) hf-2-1 (i), (**b**) hf-2-1 (ii), (**c**) hf-2-1 (iii); (**d**) hf-4-3 (i), (**e**) hf-4-3 (ii), (**f**) hf-4-3 (iii); (**g**) hf-8-1 (i), (**h**) hf-8-1 (ii), (**i**) hf-8-1 (iii).

Take hf-2-1 as an example, the failure process of hf-2-1 is presented in Figure 5a–c. During the initial loading process, the specimen remained intact, and the axial stress increased almost linearly with the development of axial strain, as shown in Figure 4a. When the axial stress reached approximately 31 MPa, there were a few vertical cracks observed on the specimen, as shown in Figure 5a. After that, more vertical cracks appeared on the specimen with the development of the loading process, and the axial stress kept increasing slowly until the second turning point, as shown in Figure 5a. After that, the axial stress experienced a short decrease and then kept rising until the peak point was reached, as shown in Figure 4a. Meanwhile, the vertical cracks kept dilating and extending toward the top and bottom sections of the specimen, as shown in Figure 5b. This feature of the stress–strain curve indicated a strain-hardening characteristic [33]. At this stage, the stress increase of the hf-2-type specimen (after the second turning point) was not as significant as that of the bf-2-type cylinder, as shown in Figure 4b. This was possibly due to the relatively low confinement strength of the HFRP spiral compared to that of the BFRP spiral with similar section stiffness, which resulted in a relatively weak constraint effect for the cylinders. When the axial strain reached approximately 0.01, a few crisp fracture sounds were captured, which were possibly due to the fracture of the HFRP spiral, as shown in Figure 5c. After that, the axial stress gradually decreased, as shown in Figure 4a, and the concrete cover gradually spalled until the concrete was crushed.

(2) hf-4 and hf-6 cylinder types

The hf-4- and hf-6-type cylinders revealed similar failure processes. Taking hf-4-3 as an example, as shown in Figure 5d–f, the failure process of hf-4-3 was similar to that of the hf-2-1 cylinder during the initial loading process before the turning point (i.e., peak stress point) was reached, as shown in Figure 4a. After the turning point, the axial stress decreased (instead of increasing as that of hf-2-1) with the development of axial strain. A strain-softening characteristic was revealed due to relatively low confinement [33]. When the axial strain reached 0.013, a crisp fracture sound was captured, possibly due to the fracture of the HFRP spiral. Then, the concrete was gradually crushed, indicating specimen failure.

(3) hf-8 cylinder type

The failure process of the hf-8 type cylinder was similar to the hf-4 (or hf-6) cylinder, as shown in Figure 5g–i. However, due to the relatively large spacing of the HFRP spiral, it failed to effectively confine the concrete core of the hf-8-type cylinder. The constraint effect of the HFRP spiral was almost negligible for the hf-8-type cylinder. As a result, the HFRP spiral remained intact throughout the loading process, and specimen failure was characterized by concrete crush, as shown in Figure 5i.

### 3.2. Strain Development Mechanism of HFRP Spiral in Concrete Cylinders

**Figure 6:**
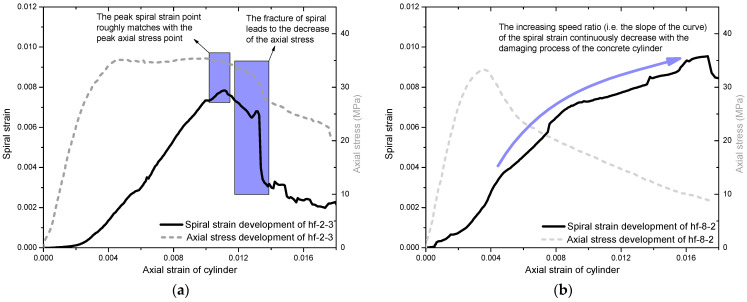
Development mechanism of spiral strain in typical cylinders: (**a**) in hf-2-3 and (**b**) in hf-8-2.

The effectiveness of the HFRP confinement could be evaluated by analyzing the strain development mechanisms in concrete cylinders. Figure 6 demonstrates the typical strain development mechanisms of the HFRP spirals embedded in the test cylinders. For a certain type, a typical compressive stress–strain curve of the cylinder, as well as a typical spiral strain curve (developed with the axial strain of the cylinder), are provided in one figure for a better comparison, as shown in Figure 6.

For well-confined cylinders (such as the hf-2-3 cylinder), the spiral strain developed slowly with the axial strain during the initial loading process due to the relatively small transverse expansion of the concrete cylinder. Once the axial strain reached 0.002, the spiral strain quickly increased with the development of the axial strain. After the axial strain reached 0.004, despite the axial stress stopping due to continuous damage of the concrete, the spiral strain could keep rising (almost linearly) with the axial strain, as shown in Figure 6a. It indicated that efficient confinement was enforced to the concrete core. Therefore, the concrete core could remain intact during this process until the failure of the HFRP spiral. When the axial strain reached around 0.011, the HFRP spiral finally fractured and, thus, triggered the failure of the concrete core. The axial stress started decreasing. This specific failure mechanism could be confirmed by comparing the peak spiral strain point to the peak axial stress point, which were roughly matched, as shown in Figure 6a. After that, the undamaged HFRP spiral section could continuously fracture and, finally, resulted in a dramatic decrease in axial stress, as shown in Figure 6a.

In contrast, insufficiently confined cylinders (such as hf-8-2) showed a distinguished development mode of spiral strain, as shown in Figure 6b. Throughout the loading process, the spiral strain of hf-8-2 could non-uniformly increase with the increase in axial strain until concrete crushing. In particular, the increasing speed rate (i.e., the slope of the curve) of the spiral strain continuously decreased with the loading process, as shown in Figure 6b. This pattern could be attributed to the insufficient confinement of the HFRP spiral failing to effectively constrain the concrete core and resulting in continuous damage to the concrete. With concrete damage accumulation, the section stiffness of the concrete cylinder continuously decreased with the loading process.

### 3.3. Ultimate Strain of HFRP Spiral

Due to the elastic properties of FRP materials, their failure in concrete structures is usually characterized by fiber fracture when the ultimate strain is reached. Since the HFRP spirals were composited with basalt fiber and carbon fiber, which have different fracture strains (i.e., elongation rates in Table 2), the ultimate strain of the HFRP spirals should be determined by their carbon fibers (i.e., the fracture strain of the HFRP spirals should equal the corresponding CFRP bars, as shown in Figure 2b, which had a relatively low elongation rate of 1.4%). However, the actual fracture strain of the HFRP spiral was larger than that of the corresponding CFRP bar, which contained the same fiber composition as the HFRP spiral, as shown in Figure 2b. This indicated an advantageous composite effect contributed by the basalt fibers. This composite effect of the HFRP spiral could be beneficial to its utilization ratio of strength.

Compared to the mechanical properties of the HFRP spiral (tested as a single rod), its performance in concrete cylinders was more crucial for the evaluation and design of the confined concrete. The recorded maximum spiral strains (*ε_fu,a_*) in different concrete cylinders, as well as the corresponding axial stresses (*f_cu,a_*) and axial strains (*ε_cu,a_*) of the cylinders, are provided in Table 4. The maximum strain of the HFRP and BFRP spirals captured in the tests equaled 0.012. The range of the maximum strain values of all the spirals was 0.007~0.012. Other than certain cylinders (hf-2 and bf-6), the maximum strain values of most cylinders ranged from 0.011 to 0.012. The maximum strain of the HFRP spirals reached over 70% of their ultimate strains (tested as a single rod), as shown in Table 4. This indicates that a relatively low strength degeneration (around 30%) was achieved for the HFRP spirals embedded in concrete cylinders. In contrast, the BFRP spirals revealed a relatively large strength degeneration (around 50%), as shown in Table 4. Note that multiple factors could affect the maximum strains of the spirals in cylinders. For instance, the disturbance of the concrete damaging process and the limited testing range of the strain gage could cause early failure of the sensors; the randomness in a spiral’s fracture point could lead to a potential underestimate, using the maximum strain of the preset SGs as the actual fracture strain of a spiral. Therefore, the actual maximum strain of a spiral could be larger than the captured maximum strain in the test, and the ultimate strength of the spirals could be underestimated.

### 3.4. Evaluation of Ultimate Stress and Strain of HFRP-Spiral-Confined Concrete Cylinders

The ultimate stress and strain of the confined concrete cylinders were crucial for their safety design and assessment. Due to the unique elastic properties of FRP materials, the performances of FRP-confined concrete cylinders are significantly different from those of steel-reinforced concrete cylinders [34,35]. Especially for the cylinders reinforced with sufficient FRP confinement, the compressive stress–strain curves revealed a unique strain-hardening characteristic, which could further enhance the ultimate strength and strain of the confined cylinders. A lot of models have been proposed for predicting the ultimate stress and strain of FRP-confined concrete [2,33,36,37,38]. A large number of them have been proposed based on experimental or theoretical studies on cylinders confined by FRP sheets, FRP strips, or FRP tubes. None of them has been proposed based on studies of FRP-spiral-confined concrete cylinders. Therefore, these models’ applicability needs to be verified for HFRP-spiral-confined concrete cylinders.

(1) Existing models

Five typical existing models were collected in this paper, as shown in Table A1, where *f_cc_′* and *ε_cc_′* are the predicted ultimate stress and strain of a confined cylinder with strain-hardening characteristics, respectively; *f_cu_′* and *ε_cu_′* are the predicted ultimate stress and strain of a confined cylinder with strain-softening characteristics, respectively; *ε_co_′* is the ultimate strain of unconfined concrete (=0.0038); *f_l_* is the confinement strength as defined in Equation (1); and *E_l_* is the confinement stiffness as defined in Equation (2).

(1) $$f_{l}=\frac{2f_{f}A_{f}}{Ds}$$

(2) $$E_{l}=\frac{2E_{f}A_{f}}{Ds}$$

Since the actual ultimate strengths of the HFRP spirals and BFRP spirals in the cylinders were obtained through the tests in this paper, the actual ultimate strengths of the spirals in the cylinders (i.e., 0.7 *f_f_* and 0.5 *f_f_* for the HFRP spiral and BFRP spiral, respectively) were applied for the value of *f_f_* in Equation (1).

The average absolute error (*Err.*) [39], as defined in Equation (3), was used to evaluate the performances of the models.

(3) $$Err.=\sum \frac{Theo._{i}-Expe._{i}}{Expe._{i}}/n$$

where *Theo._i_* and *Expe._i_* are the theoretical and experimental results of cylinder *i*, respectively; and *n* is the total number of cylinders (=3). Considering that the ultimate stress and strain of the spiral-confined cylinders usually happened after concrete cover failure, the experimental results for ultimate stress (or peak stress) were calculated with the ultimate load (or peak load) of a cylinder divided by its core area confined by the spiral (*A_cor_*), as defined in Equation (4).

(4) $$A_{cor}=\pi {\left(\frac{D_{cor}}{2}\right)}^{2}$$

where *D_cor_* is the diameter of the confined core area of the cylinder, which could be calculated with Equation (5).

(5) $$D_{cor}=D-2d_{c}-d_{f}$$

(2) Evaluation for cylinders with strain-hardening characteristics

For the cylinders revealing strain-hardening characteristics (i.e., hf-2- and bf-2-type cylinders), their ultimate stress and strain were predicted with all of the collected models. The results are listed in Table 5.

Most of the existing models overestimated the ultimate stress and strain of the FRP-spiral-confined cylinders, as shown in Table 5. This could be due to the confinement efficiency of the FRP spirals being lower than those of FRP sheets or FRP tubes. It indirectly showed a relatively low economic efficiency of the FRP spirals for the confinement of concrete. The ultimate stress errors of the hf-2-type cylinder ranged from 0.28 to 0.66, relatively higher than those of the bf-2-type cylinder (ranging from 0.14 to 0.47). The ultimate strain error revealed the same pattern as the ultimate stress error (but with a significantly larger value). It indicates that the existing models could be less accurate for predicting the performances of the HFRP-spiral-confined concrete columns than that of a common FRP-spiral-confined (such as a BFRP spiral) concrete column.

Among the collected models, Wu’s model [33] showed the most accurate prediction results. The maximum errors of Wu’s model equaled 0.28 and 0.10 for predicting ultimate stress and strain, respectively. These values were significantly lower than the maximum errors of the other models. This indicates that a model derived from the ultimate Poisson’s ratio based on the strain compatibility principle (such as Wu’s model) could be insusceptible to the confinement type of the concrete, such as FRP spiral, FRP sheet, FRP tube, etc. With more data feeding and a proper regression, Wu’s model is expected to be the most accurate model for predicting FRP-spiral-confined cylinders with strain-hardening characteristics.

(3) Evaluation for cylinders with strain-softening characteristics

For cylinders revealing strain-softening characteristics (i.e., hf-4-type, hf-6-type, bf-4-type, and bf-6-type cylinders), Wu’s model was evaluated with the test results since it was the only model that provided a prediction method for this type of cylinder (with a strain-softening characteristic). In addition, the predicted peak stress (*f_cp_′*) and peak stress (*ε_cp_′*) (as defined in Equations (6) and (7) [33], respectively, according to Wu’s model) were also evaluated with the test results. The results are listed in Table 6.

(6) $$f_{cp}{}^{\prime }=f_{co}{}^{\prime }\left(1+0.002\frac{30}{f_{co}{}^{\prime }}\frac{\rho_{f}E_{f}}{\sqrt{f_{co}{}^{\prime }}}\right)$$

(7) $$\varepsilon_{cp}{}^{\prime }=\varepsilon_{co}{}^{\prime }\left(1+0.007\frac{30}{f_{co}{}^{\prime }}\frac{\rho_{f}E_{f}}{\sqrt{f_{co}{}^{\prime }}}\right)$$

The peak and ultimate stresses predicted by Wu’s model fit relatively well with the test results. The maximum peak stress error equaled 0.07, which indicates that the peak stress of the FRP-spiral-confined cylinders (with strain-softening characteristics) could be precisely predicted. The ultimate stress error ranged from 0.07 to 0.21 (in Table 6), relatively lower than that of the cylinders with strain-hardening characteristics (ranging from 0.14 to 0.28, as shown in Table 5). This indicates that a relatively good accuracy could be obtained for predicting the ultimate stress of cylinders with strain-softening characteristics using Wu’s model. In addition, the model’s accuracy varied with the types of FRP spirals. The accuracy of the model showed a noticeable decline when comparing the HFRP-spiral-confined cylinders (hf-4 or hf-6) to the BFRP-spiral-confined cylinders (bf-4 or bf-6), as shown in Table 6. This indicates that the accuracy of Wu’s model could also decrease when applied to hybrid-FRP-spiral-confined cylinders.

Compared to the accuracy of the stress prediction, the accuracy of Wu’s model showed a significant decline when predicting the peak and ultimate strains of cylinders with strain-softening characteristics. The error ranged from 0.37 to 1.04 for predicting peak strain, which indicated a significant overestimation. Note that the predicted peak strains of the cylinders were close to their corresponding ultimate strains, as shown in Table 6, which was different from the actual strain-softening characteristics revealed in the test. Hence, the predicting method for peak strain should be rebuilt in future studies for better prediction.

## 4. Conclusions

An experimental study on the compressive behavior of concrete cylinders confined by novel HFRP spirals was provided in this paper. The compressive behaviors of HFRP-spiral-confined concrete cylinders were comparatively analyzed and discussed. The following conclusions could be drawn:

(1) The compressive failure mode and mechanism of the HFRP-spiral-confined cylinders were similar to those of BFRP-spiral-confined cylinders, indicating that the design method of HFRP-spiral-confined concrete could be similar to that of common FRP-spiral-confined cylinders.
(2) The ultimate strain of the HFRP spiral (tested as a single rod) was larger than that of the corresponding CFRP bar. This proved that an advantageous composite effect could be obtained with the HFRP spiral. The maximum strain of the HFRP spirals in concrete cylinders reached over 70% of the ultimate strain values, relatively larger than that of the BFRP spirals, indicating that an ideal in-service strength could be obtained with the HFRP spiral.
(3) Most of the existing models overestimated the ultimate stress and strain of the FRP-spiral-confined cylinders. Wu’s model, derived from the ultimate Poisson’s ratio based on the strain compatibility principle, gave the most accurate prediction, both for cylinders with strain-hardening and for those with strain-softening characteristics.

## Figures and Tables

**Table 1:** Design details and test results of cylinders.

Cylinder Type	FRP Spiral			Concrete Cylinders		
	Type	*s* (mm)	*ρ_f_* (%)	Failure Mode	*f_cp_* (or *f_cc_*) (MPa)	*ε_cp_* (or *ε_cc_*)
pc	\	\	\	CC	44	0.004
hf-2	HFRP spiral	23	4.0	SF	36 (*f_cc_*)	0.011 (*ε_cc_*)
hf-4	HFRP spiral	46	2.0	SF	34	0.005
hf-6	HFRP spiral	69	1.3	SF	32	0.005
hf-8	HFRP spiral	92	1.0	CC	32	0.004
bf-2	BFRP spiral	23	7.6	SF	41 (*f_cc_*)	0.013 (*f_cc_*)
bf-4	BFRP spiral	46	3.8	SF	34	0.005
bf-6	BFRP spiral	69	2.5	SF	32	0.005
bf-8	BFRP spiral	92	1.9	CC	31	0.004

Note: CC = concrete crush failure; SF = spiral fracture failure.

**Table 2:** Mechanical and physical properties of spiral components.

Component Type	Density (g/cm^3^)	Diameter (μm)	Tensile Strength (MPa)	Elastic Modulus (GPa)	Elongation Rate (%)
basalt fiber	2.63	13	2250	90	2.5
carbon fiber	1.85	6	3000	210	1.4
vinyl epoxy resin	1.06	—	95	3.6	6.1

**Table 3:** Physical and mechanical properties of reinforcements.

Spiral Type	Fiber Type	*d_f_* (mm)	*A_f_* (mm^2^)	*f_f_* (MPa)	*E_f_* (GPa)	*ε_fu_*	*E_f_*·*A_f_* (GPa·mm^2^)
HFRP spiral	basalt and carbon	5.8	26.4	1667	101	0.016	2667
BFRP spiral	basalt	7.9	49.0	1281	53	0.024	2597

**Table 4:** Recorded maximum spiral strains in different concrete cylinders.

Cylinder Type	*ε_fu,a_*	*ε_fu,a_*/*ε_fu_* (%)	*f_cu,a_*	*ε_cu,a_*	Cylinder Type	*ε_fu,a_*	*ε_fu,a_*/*ε_fu_* (%)	*f_cu,a_*	*ε_cu,a_*
hf-2	0.008	49%	36	0.011	bf-2	0.012	49%	41	0.013
hf-4	0.012	73%	30	0.006	bf-4	0.012	51%	28	0.010
hf-6	0.011	72%	23	0.011	bf-6	0.007	28%	24	0.012
hf-8	0.011	70%	9	0.017	bf-8	0.011	47%	8	0.018

**Table 5:** Evaluation of existing models for predicting the ultimate stress and strain of cylinders with strain-hardening characteristics.

Model	hf-2				bf-2			
	*f_cc_^′^* (MPa)	*Err.*	*εε_cc_^′^*	*Err.*	*f_cc_^′^* (MPa)	*Err.*	*εε_cc_^′^*	*Err.*
Lam and Teng (2003) [36]	103	0.66	0.037	2.68	104	0.47	0.038	1.94
Wu et al. (2006) [33]	80	0.28	0.011	0.10	80	0.14	0.012	−0.08
Teng et al. (2009) [37]	100	0.61	0.030	2.03	102	0.45	0.032	1.48
Wei and Wu (2012) [38]	84	0.34	0.025	1.49	84	0.19	0.025	0.94
Yu-Fei Wu et al. (2015) [2]	86	0.38	0.021	1.12	87	0.23	0.022	0.70

**Table 6:** Evaluation of Wu’s model [33] for predicting the performances of cylinders with strain-softening characteristics.

Cylinder	*f_cp_^′^* (MPa)	*Err.*	*ε* *_cp_* *^′^*	*Err.*	*f_cu_^′^* (MPa)	*Err.*	*ε* *_cu_* *^′^*	*Err.*
hf-4	62	0.07	0.009	1.04	55	0.07	0.010	0.63
hf-6	56	0.03	0.008	0.58	48	0.21	0.008	−0.26
bf-4	62	0.02	0.009	0.78	56	0.11	0.010	−0.01
bf-6	56	−0.01	0.007	0.37	48	0.12	0.008	−0.31

## Data Availability

The data presented in this study are available on request from the corresponding author.

## Appendix A

**Table A1:** Existing models of FRP-confined concrete evaluated in this paper.

Model	Ultimate Strength	Ultimate Strain
Lam and Teng (2003) [36]	$\frac{f_{cc}{}^{\prime }}{f_{co}{}^{\prime }}=1+3.3\frac{f_{l}}{f_{co}{}^{\prime }}$	$\frac{\varepsilon_{cc}{}^{\prime }}{\varepsilon_{co}{}^{\prime }}=1.75+12\frac{f_{l}}{f_{co}{}^{\prime }}{\left(\frac{\varepsilon_{fu,a}}{\varepsilon_{co}{}^{\prime }}\right)}^{0.45}$
Wu et al. (2006) [33]	$\frac{f_{cc}{}^{\prime }}{f_{co}{}^{\prime }}=1+2.0\frac{f_{l}}{f_{co}{}^{\prime }}$(strain hardening) $\frac{f_{cu}{}^{\prime }}{f_{co}{}^{\prime }}=0.75+2.5\frac{f_{l}}{f_{co}{}^{\prime }}$(strain softening)	$\varepsilon_{cc}{}^{\prime }=\frac{\varepsilon_{fu,a}}{v_{u}};v_{u}=0.56{\left(\frac{f_{l}}{f_{co}{}^{\prime }}\right)}^{-0.66}$ $\frac{\varepsilon_{cu}{}^{\prime }}{\varepsilon_{co}{}^{\prime }}=1.3+6.3\frac{f_{l}}{f_{co}{}^{\prime }}$
Teng et al. (2009) [37]	$\frac{f_{cc}{}^{\prime }}{f_{co}{}^{\prime }}=1+3.5\left[\left(\frac{E_{l}}{f_{co}{}^{\prime }/\varepsilon_{co}{}^{\prime }}\right)-0.01\right]\left(\frac{\varepsilon_{fu,a}}{\varepsilon_{co}{}^{\prime }}\right);\frac{\varepsilon_{fu,a}}{\varepsilon_{co}{}^{\prime }}\geq 0.01$ $\frac{f_{cc}{}^{\prime }}{f_{co}{}^{\prime }}=1;\frac{\varepsilon_{fu,a}}{\varepsilon_{co}{}^{\prime }}<0.01$	$\frac{\varepsilon_{cc}{}^{\prime }}{\varepsilon_{co}{}^{\prime }}=1.75+6.5{\left(\frac{E_{l}}{f_{co}{}^{\prime }/\varepsilon_{co}{}^{\prime }}\right)}^{0.8}{\left(\frac{\varepsilon_{fu,a}}{\varepsilon_{co}{}^{\prime }}\right)}^{1.45}$
Wei and Wu (2012) [38]	$\frac{f_{cc}{}^{\prime }}{f_{co}{}^{\prime }}=0.5+2.7{\left(\frac{f_{l}}{f_{co}{}^{\prime }}\right)}^{0.73}$	$\frac{\varepsilon_{cc}{}^{\prime }}{\varepsilon_{co}{}^{\prime }}=1.75+12{\left(\frac{f_{l}}{f_{co}{}^{\prime }}\right)}^{0.75}{\left(\frac{30}{f_{co}{}^{\prime }}\right)}^{0.62}$
Yu-Fei Wu et al. (2015) [2]	$\frac{f_{cc}{}^{\prime }}{f_{co}{}^{\prime }}=0.75+2.7{\left(\frac{f_{l}}{f_{co}{}^{\prime }}\right)}^{0.9}$	$\frac{\varepsilon_{cc}{}^{\prime }}{\varepsilon_{co}{}^{\prime }}=1.75+140\left(\frac{f_{l}}{f_{co}{}^{\prime }}\right){\left(\varepsilon_{fu,a}\right)}^{0.6}$
